# Seasonal Variation in Soil and Herbage CO_2_ Efflux for a Sheep-Grazed Alpine Meadow on the North-East Qinghai-Tibetan Plateau and Estimated Net Annual CO_2_ Exchange

**DOI:** 10.3389/fpls.2022.860739

**Published:** 2022-06-02

**Authors:** Hang Yuan, Cory Matthew, Xiong Zhao He, Yi Sun, Yang Liu, Tao Zhang, Xiaoye Gao, Caiyu Yan, Shenghua Chang, Fujiang Hou

**Affiliations:** ^1^Key Laboratory of Grassland Livestock Industry Innovation, Ministry of Agriculture and Rural Affairs, College of Pastoral Agriculture Science and Technology, Lanzhou University, Lanzhou, China; ^2^School of Agriculture and Environment, Massey University, Palmerston North, New Zealand

**Keywords:** carbon cycle, carbon sequestration, *Kobresia*, grazing, metabolisable energy

## Abstract

The Qinghai-Tibetan Plateau is a vast geographic area currently subject to climate warming. Improved knowledge of the CO_2_ respiration dynamics of the Plateau alpine meadows and of the impact of grazing on CO_2_ fluxes is highly desirable. Such information will assist land use planning. We measured soil and vegetation CO_2_ efflux of alpine meadows using a closed chamber technique over diurnal cycles in winter, spring and summer. The annual, combined soil and plant respiration on ungrazed plots was 28.0 t CO_2_ ha^−1^ a^−1^, of which 3.7 t ha^−1^ a^−1^occurred in winter, when plant respiration was undetectable. This suggests winter respiration was driven mainly by microbial oxidation of soil organic matter. The winter respiration observed in this study was sufficient to offset the growing season CO_2_ sink reported for similar alpine meadows in other studies. Grazing increased herbage respiration in summer, presumably through stimulation of gross photosynthesis. From limited herbage production data, we estimate the sustainable yield of these meadows for grazing purposes to be about 500 kg herbage dry matter ha^−1^ a^−1^. Addition of photosynthesis data and understanding of factors affecting soil carbon sequestration to more precisely determine the CO_2_ balance of these grasslands is recommended.

## Introduction

The rise in global human population during the latter half of the 20th century, and continuing at the present time, has sparked a wide range of studies aimed at defining human impact on the environment and mitigating those impacts likely to result in detrimental future change (IPCC, 2013). One of the key questions moving forward into the 21st century is how to minimize the environmental footprints of food production systems, such as release of greenhouse gases. The main greenhouse gas (GHG) present in the atmosphere is CO_2_, which is estimated to be responsible for 60% of the anthropogenic greenhouse effect (Rodhe, 1990). There has been a well-documented rise in atmospheric CO_2_ concentration from approximately 315 parts per million (ppm) in 1960 to over 410 ppm in 2020, and this rise is ongoing. Grasslands of the world are estimated to account for 40.5% of the terrestrial land area excluding Greenland and Antarctica (Reynolds, 2005) and are potentially important to managing global atmospheric CO_2_ concentration. Hence, a keen interest is developing to better understand ecological processes in grasslands such as carbon cycling. This understanding is expected to provide tools for planning land use at a national level, to enhance sustainability of grassland ecosystems and to maximize delivery to the population of ecosystem services to the population, such as food supply, recreational opportunity, biodiversity maintenance and climate regulation while at the same time enhancing sustainability of the grassland ecosystems providing those services.

For carbon cycling of an ecosystem the basic equation to describe the major components and their interrelationship is:


(1) $$NEE=GPP+R_{e}$$

where NEE is net ecosystem exchange, GPP is gross primary production representing photosynthetic CO_2_ capture, and R_e_ is the ecosystem respiration. NEE is primarily comprised of vegetation-related fluxes including net photosynthesis but can include a range of other components such as the impact of herbivores. Re can be further partitioned into above- and below-ground components (R_ea_ and R_eb_, respectively) and R_ea_ and R_eb_ can be further separated into autotrophic and heterotrophic contributions, with heterotrophic R_eb_ including the contribution of the soil microbial community. Hence, respiration associated with vegetation cover, (i.e., the autotrophic component of R_e_), is an important component of global carbon cycling and CO_2_ emissions to the atmosphere. The most common method for measuring NEE is eddy covariance (EC). However, EC data for NEE provides only the sum of GPP and R_e_ without directly measuring either of them. To resolve the components of NEE, R_e_ is often estimated from temperature data or GPP from solar radiation data. Since both of these approaches involve major assumptions, directly measured R_e_ (and associated soil temperature data) are valuable complementary data to assist with interpretation of NEE data obtained by EC. Soil respiration over an annual cycle (i.e., R_eb_) was reported in one major study aggregating data of many forest ecosystems across Europe to be approximately 60% of gross aboveground primary production (GAPP; Janssens et al., 2001). A synopsis of the major components of the global carbon cycle by Rustad et al. (2000), also highlighted the importance of soil respiration but noted that there are technical difficulties and high errors when measuring it, and therefore a knowledge gap in this area. We are not aware of a comparable compilation of grassland data but a first expectation would be that soil respiration in grassland would be a similar or higher proportion of GAPP. These authors considered it likely that global warming will generally increase soil respiration, so releasing more CO_2_ and further increasing global warming. In another review of the global carbon cycle directed at informing policymakers, Schlesinger and Andrews (2000) identified the ratio of soil respiration: GAPP as 75:120 (62.5%) and stated that “nearly all models of global climate change predict a loss of carbon from soils as a result of global warming,” and note that the response is greatest in soils in cold climates. It is indicative of the difficulties measuring the contribution of soil respiration to ecosystem carbon cycling, that almost a decade after these two reviews, a European study referred to the potential for C sequestration by world grasslands as “speculative,” and of “uncertain quantitative importance” (Rogiers et al., 2008). That particular study demonstrated that a mountain hay meadow ecosystem studied in Switzerland was a net source of CO_2_ over a 3-year study period from 2002 to 2005 (1.2–2.6 t C ha^−1^ a^−1^) and that losses were increased by grazing and by snow cover in winter. The sustained release of CO_2_ in these alpine meadows was attributed to ongoing oxidation of soil carbon as a consequence of drainage that had been carried out around 1940. Elsewhere, a 2-year study of the growing-season carbon balance in natural *Miscanthus sinensis* grassland in Japan (Toma et al., 2011) was unable to determine with certainty if the site studied was a net source or sink for CO_2_ on an annual basis.

These uncertainties from earlier studies highlight a general need for additional insight into the R_e_ contribution to C cycling and C source-sink status of grasslands, and Chinese grasslands are an ideal “model system” for such a study. In China, the steppe grasslands and alpine meadows of Inner Mongolia and the Qinghai-Tibetan Plateau cover a total area of some 240 million ha, excluding the more extreme desert environments. On the Qinghai-Tibetan plateau the prevailing cold climate predisposes to formation of dark soils with high organic matter content and a mollic epipedon. For one of these soils, Yuan and Hou (2015) reported soil organic C contents >12% at sites with lower historic grazing intensity and reducing to 4%–5% under sustained intensive grazing. Estimates of the carbon storage of Qinghai-Tibetan Plateau grassland soils vary but range from 6.5 to 21.4 kg C m^−2^ and 7.4–35.4 Pg total C stock (Fang et al., 2010). In one study of alpine meadow grassland on the Tibetan Plateau, grazing exclusion for three or 5 years increased NEE by 47.37% and 15.84%, respectively, and increased growing season carbon sequestration accordingly, compared with a free grazing treatment (Chen et al., 2015). With China’s large population of 1.4 billion in 2020, there is now wide recognition that these extensive grassland ecosystems will have an important role in sustainable support of the human population in future. Therefore, more information and good contextualization of that research is needed in order to position the pastoral industry in this geographically large area for optimal contribution of ecosystem services to the human population and for formulation of C budgets and definition of the global contribution to greenhouse gas cycling.

Here, we present data on soil-to-atmosphere CO_2_ fluxes directly measured using the closed chamber technique in a botanically diverse alpine grassland meadow typical of mid-altitude localities on the Qinghai-Tibetan plateau. Our study was designed to quantify both diurnal and seasonal change in the respiration components of ecosystem CO_2_ flux, R_ea_ and R_eb_, and how these fluxes respond to sheep stocking rate (The sum of R_ea_ + R_eb_ would be estimated by measurements on intact herbage while R_eb_ would be estimated by measurement of an adjacent area with herbage removed at ground level, and R_ea_ found by difference.). These data will complement those available from eddy covariance studies where R_ea_ and R_eb_ are seldom measured directly and separately and where alternative grazing managements are seldom compared because of scale factors implicit in the eddy covariance technique. We also measured soil microbial biomass, which may allow some inference about the contribution of heterotrophic soil microorganisms to R_eb_ under different sheep stocking rates. Our hypothesis was that with warming climatic conditions and increasing animal numbers on the Qinghai-Tibetan plateau during the last 30 years, soil respiration losses of C from the ecosystem may be increasing as predicted in the European literature cited above and may be now larger than generally realized. We wished to quantify the current C-respiration losses in order to provide data for future C-balance determinations for these alpine meadows.

## Experimental

### Study Site

**Figure 1:**
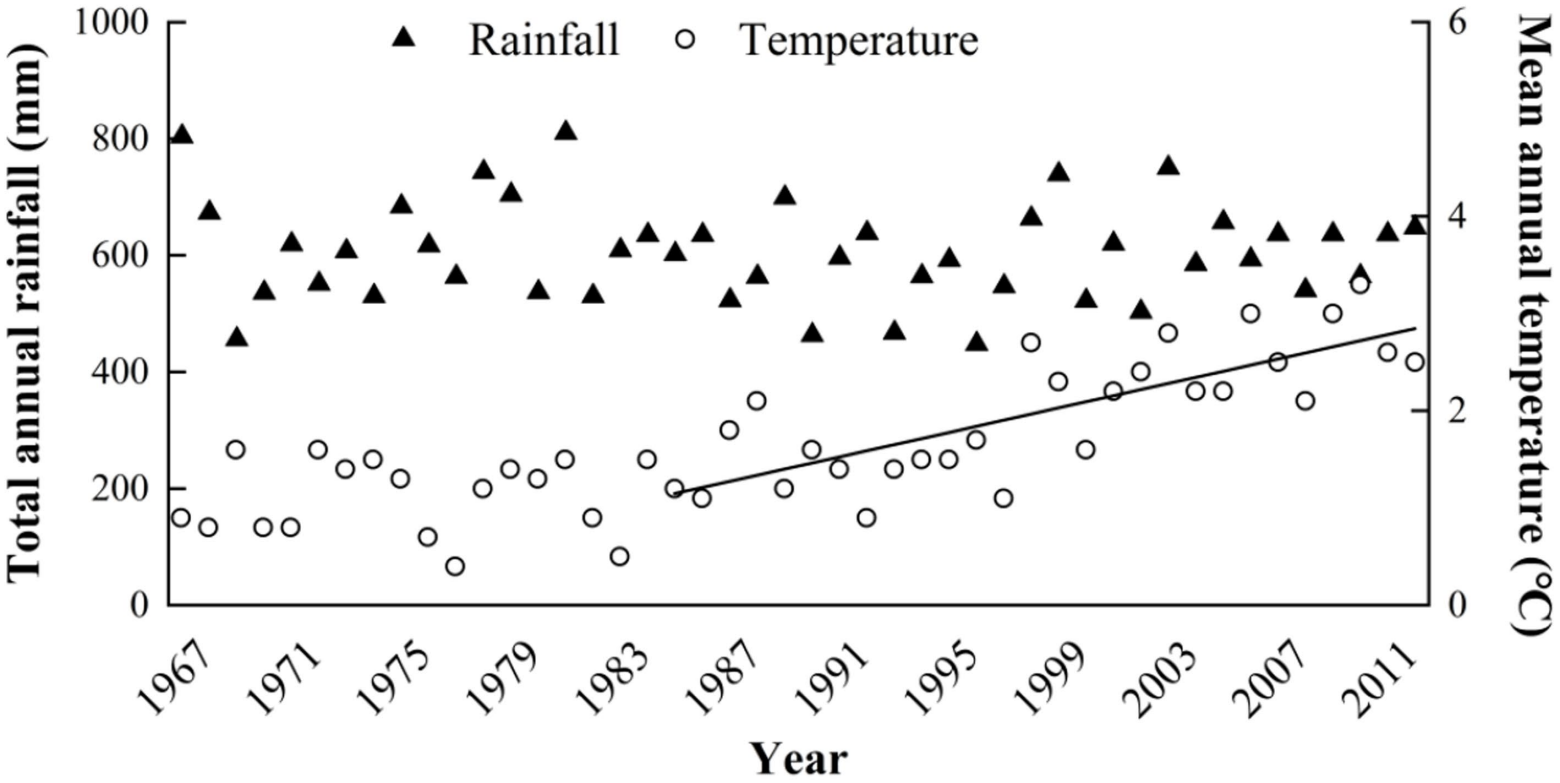
Data for annual precipitation and annual mean temperature (AMT), 1967–2012 provided by the Gansu Meteorological Bureau for Maqu County, Tibet, where the experiment was located. Equation for Regression of mean annual temperature (MAT) on year was (taking 1967 as year zero): MAT = 1.09 + 0.0027 (SE + 0.0148) °C per year (NS) for the 20-year period 1967–1986 and MAT = 0.094 + 0.0637 (SE + 0.0090) °C per year (*p* < 0.001) for the 30-year period 1983–2012 (as indicated by the trend line).

The study was conducted on a 20 ha, botanically diverse, fenced alpine meadow located on the eastern Qinghai-Tibetan Plateau at the Maqu County Azi Livestock Breeding Base (Lat. 35°58′N, 101°53′E; 3,750 m elevation) in Gansu Province, People’s Republic of China. This area is classified as alpine humid grassland (alpine meadow), with a frost-free period of 90–100 days, an annual mean air temperature of 1.2°C, and monthly mean maximum 11.7°C in July and minimum of −10 in January. The mean annual precipitation of approximately 620 mm is distributed unevenly among seasons, primarily falling as rain during the short, cool summer (Table 1). The annual cloud-free sunshine is about 2,580 h. Inter-annual variation in temperature and rainfall for the previous 45 years is shown in Figure 1 and time trends for this data are evaluated in “Maqu County Climate Data” section below. The soils at the experiment site are well drained so accumulation of excess soil moisture should not have been a major factor in determining respiration responses we measured.

**Table 1:** Seasonal variation in mean monthly temperature (°C) and monthly precipitation (mm) for Maqu County where the experiment was located.

	Jan	Feb	Mar	Apr	May	Jun	Jul	Aug	Sep	Oct	Nov	Dec
Mean monthly temperature	−8.0	−4.9	−1.4	3.3	6.5	9.6	12.0	11.3	8.4	2.9	−3.2	−4.9
Total monthly precipitation	6.0	5.3	13.2	26.3	68.0	104.0	141.7	101.7	90.8	46.2	7.3	1.6

Data are averages for a 14 year period 1999–2012 and were obtained from the Gansu Meteorological Bureau.

### Grazing Management and Herbage Intake of Animals

The experiment was set up as a lamb production system with lambs purchased at 6 months of age in late May and sold for slaughter in late December of each year, commencing in 2010. Two visually homogeneous areas located about 300 m apart on a high alluvial plain were selected for July to September (summer) and October to December (winter) grazing and fenced to provide four replicate plots sized 100 × 100 m (low stocking rate) and four further plots sized 100 × 50 m (high stocking rate), in both the summer and winter grazing areas. In each of the 16 plots created in this way, a 10 × 10 m sub-plot was fenced so samples from ungrazed pasture could be collected for pairwise comparison with data from the grazed pastures. All plots were stocked with eight sheep and rotationally grazed. For grazing, each paddock was divided into three strips of equal area and the eight sheep spent 10 days on each strip. The low and high stocking rates therefore involved the equivalent of 1.97 or 3.95 lambs ha^−1^ continuously stocked during daylight hours, but with sheep housed at night. Three 30-day cycles were completed in this way on both the summer and the winter grazing areas. However, for simplicity we will refer to the grazing treatments in terms of animals per unit area during grazing of plots: 8 and 16 sheep ha^−1^, respectively. Animals were weighed monthly and herbage consumed by the animals was estimated based on energy requirements of the animals, as described by Chen et al. (2010).

Our animal night-housing facility is of a standard design used in China and conforms to the national standard “Laboratory Animals Environment and Housing Facilities” (GB 14925-2010), the care of animals during the experiment conformed to relevant Chinese government protocols.

### CO_2_ Efflux

The soil-atmosphere CO_2_ fluxes in the field were measured by a static closed chamber method (Levy et al., 2011). Two identical cube-shaped chambers (40 × 40 × 40 cm) were constructed from stainless steel. The chambers were sheathed in foam plastic for improved temperature stability and fitted with an internal fan to ensure complete gas mixing and with a port with a septum for gas sampling. For a gas seal at the soil surface, the bottom edge of each chamber was seated into a water filled gas flux barrier collar inserted to a depth of 7 cm into the soil and protruding 2 cm above the soil surface. Gas sampling was performed with plastic syringes (20 ml capacity) fitted with three-way stopcocks and connected to multilayer foil/plastic sampling bags commercially available in China for storing gas samples collected for research purposes (Dalian Delin Gas Packaging Co. Ltd.). At collection, samples were withdrawn into the syringe and then, after switching the stopcock, injected immediately into the plastic gas sampling bags. Samples were analyzed within 12 h by a DLT-100 Greenhouse Gas Analyzer (GGA) with syringe injection (Model 908-0011). During gas sampling, two chambers were normally installed about 1 m apart in the same plot, one with herbage intact and one with herbage clipped to expose bare soil. High and low stocking rate plots in each replicate were sampled simultaneously, thus utilizing the four chambers. Grazed and ungrazed partitions of each plot were sampled on successive days. For logistical reasons, collar placement and clipping of herbage to ground level occurred the day before gas flux measurement. Each time the chamber collars were installed on a new plot, any dung inside the perimeter was removed by hand before closing the chamber. In each sampling “run” after a chamber was placed into its collar, four gas samples were collected 0, 10, 20, and 30 min after closing the chambers. Soil temperature (5 cm depth) inside the chamber at the start of each sampling run and chamber air temperature at the start and end of each sampling run were recorded. After collars were placed in a plot, sampling runs were carried out at 4:00, 8:00, 10:00, 12:00, 14:00, 16:00, 18:00, 20:00, and 24:00 h for each plot, without moving the collars, to define diurnal changes in gas flux. Thus, a complete seasonal measurement cycle of four grazed plots and their ungrazed partitions required 288 runs: (high or low stocking rate) x (intact vegetation or bare soil) x (the diurnal cycle of nine sampling times) x (grazed or ungrazed) x (four replicates). Gas sampling was carried out in winter (late November to early December 2010), in spring (late April to early May 2011) and in the summer growing season (late July to early August 2011). For spring and summer samplings the number of chamber runs was reduced from 288 to 216 by sampling only one of the two ungrazed control partitions in each replicate of high and low stocking rate treatments. Each seasonal set of 288 or 216 sampling runs was completed within 14–21 days.

CO_2_ gas concentration changes in the chamber were determined by regression analysis of concentration on time (see below) with units of ppm min^−1^ using the equation:


(2) $$F=\rho \frac{V}{A}\frac{P}{P_{0}}\frac{T_{0}}{T}\frac{dC_{t}}{d_{t}}$$

Where: *F* = gas flux (mg m^−2^ h^−1^), *ρ* = gas density at 273°K and 1 atmosphere pressure (kg m^−3^), *V* = chamber volume (m^3^), *A* = soil contact area of the chamber (m^2^), *P* = measurement pressure (for Tibet ~67 kPa), *P*_0_ = reference pressure (1 atmosphere, 101.3 kPa), *T*_0_ = reference temperature (273°K), *T* = measurement temperature (°K), d*C_t_*/d*_t_* = rate of change of gas concentration in the chamber (ppm min^−1^). From Eq. 2, assuming a CO_2_ density of 1.902 kg m^−3^ at 280°K and 100 kPa pressure, an internal chamber height of 42 cm including the seating channel, and further assuming a temperature and pressure of 5°C and 67 kPa, respectively (typical of average conditions on the Qinghai-Tibetan plateau), then 1 ppm min^−1^ CO_2_ flux in the chamber used in this research = 32.46 mg m^−2^ h^−1^ CO_2_ flux from an ecosystem perspective.

To scale up from plot scale to annual fluxes a weighted average of diurnal fluxes was calculated for each season and winter values assumed for months November to March, spring values for months April to June and summer values for months July to October. When performing these calculations, temperature response coefficients from regression analysis as described below were used to adjust CO_2_ flux data for the measured temperature difference between bare soil and soil with vegetation intact (Li and Sun, 2011).

### Herbage Mass

Biomass change between the start and end of grazing in the summer grazing plots in 2011 was measured by cutting, drying and weighing herbage from four 0.25 m^2^ quadrats in each plot. Herbage mass data were not collected from winter grazing plots in 2010 but were collected when identical grazing management treatments were imposed in late 2011.

### Microbial Biomass Determination

Soil samples were collected from each plot in conjunction with gas sampling in winter 2010 and spring and summer 2011. The microbial biomass fraction of soil C was determined using a chloroform fumigation extraction method (Oren et al., 2018). Briefly, organic C was extracted from fumigated and unfumigated soil samples using 0.5 M K_2_SO_4_ solution, and the extract passed through standard filter paper, and vacuum-filtered with 0.45 μm millipore filters to remove particulate matter. Organic carbon in solution was then oxidized using a known quantity of a K_2_Cr_2_O_7_/H_2_SO_4_ reagent, and the unused oxidizing reagent quantified by back-titration with Fe_2_SO_4_ solution and phenanthroline as a redox indicator to determine the titration endpoint, as described by Fan et al. (2013). Soil microbial biomass C was calculated as 2.64 × the difference in soil organic C between fumigated and unfumigated soil samples, implying an assumption that 38% of microbial biomass C had been evolved during incubation following fumigation (Oren et al., 2018).

### Statistical Analysis

A multiple regression analysis using Type III sums of squares in the GLM procedure of SAS (SAS Institute 2011) was employed to analyse the data. For each of the three seasons separately, the gas concentration data for CO_2_ measured in the chambers (1,152, 864 and 864 data points in winter, spring and summer, respectively, accumulated across the measurement cycles in each season) were regressed on minutes since chamber closing to give a regression slope representing the rate of CO_2_ accumulation inside the chamber in ppm min^−1^. Terms for the interaction between “min” (minutes since chamber closure) and other experiment treatment factors were added to the model to evaluate the change in rate of CO_2_ efflux for intact vegetation versus bare soil, the slope difference associated with grazing intensity, and for variation in air or soil temperature. The SAS model used for the combined data set in each season can be represented as follows:


(3) $$\begin{array}{l} PPM_{ct}=Ambient+a.Temp+b.PPM/\min\;SOIL\\ +c.PPM/\min\;HERB+d.PPM/\min\;GRAZ \end{array}$$

where PPM*_ct_* denotes the measured CO_2_ concentration (ppm) in the chamber at *t* minutes (0, 10, 20, or 30) from chamber closing, Ambient is the regression intercept representing measured CO_2_ concentration at chamber closing (*t* = 0 min), Temp is chamber temperature during the measurement run, PPM.min^–1^ SOIL is the contribution of soil respiration in ppm min^−1^ to PPM*_ct_* (estimating R_eb_), PPM.min^−1^ HERB is the contribution of herbage respiration in ppm min^−1^ to PPM*_ct_* (estimating R_ea_), PPM.min^−1^ GRAZ is the effect of grazing intensity on PPM*_ct_* in units of ppm min^−1^ (sheep/ha)^−1^, and *a*, *b*, *c*, and *d* are parameters estimated by the multiple regression.

A model including a term for hour of the day was also evaluated. Because slope coefficients in a multiple regression can be affected in complex ways by correlations between experimental factors such as air temperature and soil temperature, we examined the impact of air and soil temperature on CO_2_ flux in separate runs of the SAS model. Separately from the full model described above, we also performed regression analyses to determine the linear and quadratic responses of CO_2_ efflux to temperature for bare soil and intact herbage, averaged over grazing intensity effects. For the purposes of obtaining coefficients for scaling up from the experiment to the ecosystem level, weighted averages of slope coefficients for individual gas flux measurement runs for time periods of interest were also calculated where statistical significance had been confirmed by multiple regression analysis as described above.

## Results

### Maqu County Climate Data

Visual inspection of climate data (Figure 1) suggested a warming trend for mean annual temperature (MAT), beginning in the mid 1980s. This was statistically tested by linear regression. For the arbitrary 20-year period 1967–1986 the linear regression equation was (taking 1967 as year zero): MAT = 1.09 + 0.0027 (SE ± 0.0148) °C year^−1^ (NS). However, for the 30-year period 1983–2012 (a standard 30-year climate evaluation cycle), the equation was MAT = 0.094 + 0.0637 (SE ± 0.0090) °C year^−1^ (*p* < 0.001).

### Diurnal Temperature Ranges During Gas Flux Measurement

**Figure 2:**
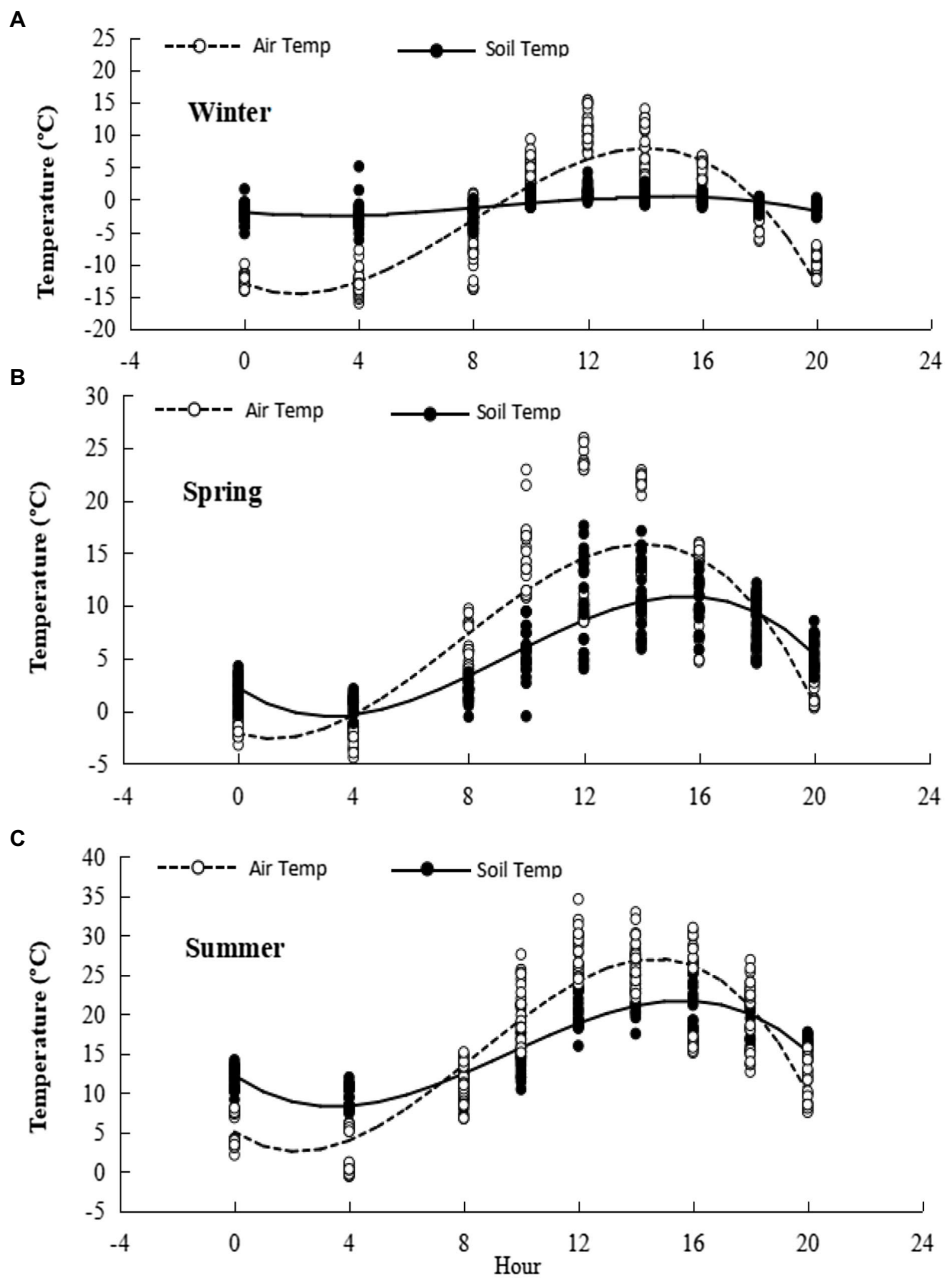
Diurnal temperature ranges during gas flux measurement. Cubic equations for fitted diurnal trends are presented in Supplementary Table S1. **(A)** Winter, **(B)** Spring, **(C)** Summer.

The diurnal range of air temperature recorded in the chambers during the three measurement cycles was wide and the daily temperature peak was of short duration. For the three seasons, maximum, minimum and mean air temperatures, respectively, were: summer, 33.0, 10.0, and 16.9°C; winter, 14.8, −13.2, and −2.4°C; spring, 25.6, −2.1, and 8.0°C. The diurnal range of soil temperature was much less then for air temperature. Notably, mean winter soil temperature averaged exactly 0°C, with midday temperatures often above freezing (Figure 2).

### CO_2_ Fluxes From Soil and Herbage Respiration

**Figure 3:**
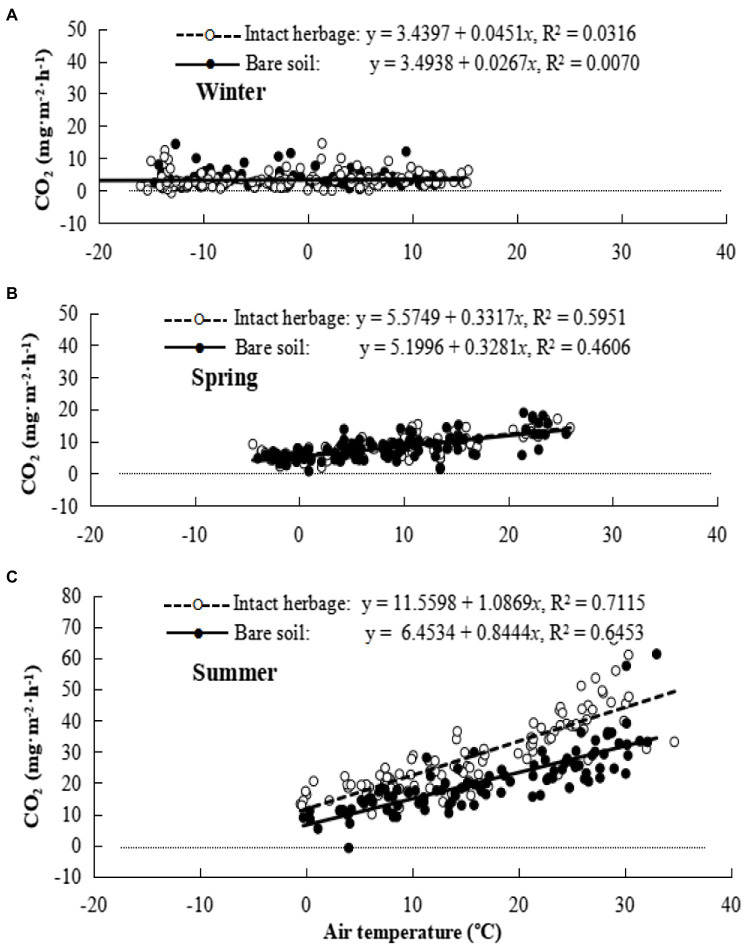
Seasonal carbon dioxide (CO_2_) emission for bare soil and intact herbage. **(A)** Winter, **(B)** Spring, **(C)** Summer.

Seasonal changes in the temperature effects on rate of CO_2_ efflux from bare soil (filled circles) and soil with intact vegetation (hollow circles) are shown in Figure 3. Notably, the multiple regression coefficient for bare soil respiration at 0°C was relatively constant over the seasons and indicated “background” respiration occurring in winter at a rate approximately 50% of that in spring and summer. However there was seasonal variation in the response of respiration rate to temperature, with a very small but statistically significant negative response to temperature increase in winter, a small positive response to temperature increase in spring, and a much larger response to temperature increase in summer. When quadratic curves were fitted to the respiration response to temperature, the quadratic coefficient was significantly positive in spring and summer but not winter (Table 2). Herbage respiration was statistically undetectable in winter, small in spring, and was the dominant contribution to respiration in summer (Figure 3; Table 2). Fluxes of CO_2_ were statistically more strongly correlated with air temperature than soil temperature in winter, but air and soil temperature correlated equally well with CO_2_ flux in spring and summer.

**Table 2:** Statistical significance (F-statistic from regression analysis and value of *p*) of terms and multiple regression coefficients for GLM analysis of CO_2_ efflux measured by a chamber technique.

	Winter	Spring	Summer
*Statistical significance of terms, full model; values are the F-statistic from regression with value of p in parentheses*			
CO_2_ efflux (i.e., respiration) of bare soil	191.76 (<0.0001)	525.82 (<0.0001)	105.98 (<0.0001)
Air temperature effect on CO_2_ efflux	7.50 (0.0063)	483.64 (<0.0001)	380.22 (<0.0001)
Soil temperature effect on CO_2_ efflux^a^	ns	503.15 (<0.0001)	330.67 (<0.0001)
Herbage contribution to CO_2_ efflux	ns	5.21 (0.0227)	130.48 (<0.0001)
Grazing intensity effect on CO efflux^b^	ns	ns	20.26 (<0.0001)
R-squared (*p*)	0.204 (<0.0001)	0.748 (<0.0001)	0.725 (<0.0001)
*Multiple regression co-efficients, full model (Values of p for coefficients are identical to those of their F-values above.)*			
Intercept (ppm CO_2_)	424.2	410.7	394.7
CO_2_ efflux of bare soil at 0°C (R_eb_, ppm min^−1^)^c^	2.777	5.411	5.814
Herbage contribution to CO2 efflux (R_ea_ ppm min^−1^)	−	0.487	7.816
Air temperature effect on CO_2_ efflux (ppm min^−1^°C^−1^)	−0.033	0.295	0.726
Soil temperature effect on CO_2_ efflux (ppm min^−1^°C^−1^)^a^	−	0.535	1.293
Grazing intensity effect (ppm min^−1^/sheep ha^−1^)^b^	−	−	0.0078
*Quadratic regression model analyzing only for temperature effect on ppm min^−1^ CO_2_ efflux after chamber closure*			
Intercept (ppm CO_2_)	424.2 (<0.0001)	410.7 (<0.0001)	394.9 (<0.0001)
CO_2_ efflux of bare soil (ppm min^−1^)^a^	2.961 (<0.0001)	5.959 (<0.0001)	14.97 (<0.0001)
Air temperature linear coefficient	−0.0295 (0.0240)	0.1368 (<0.0001)	0.1465 (ns)
Air temperature quadratic coefficient	0.0005 (ns)	0.0080 (<0.0001)	0.0173 (0.0003)
R-squared (*p*)	0.203 (<0.0001)	0.754 (<0.0001)	0.681 (<0.0001)
*Linear regression model analyzing only for temperature effect on ppm min^−1^ CO_2_ efflux after chamber closure*			
Intercept (ppm CO_2_)	424.2	410.9	394.7
CO_2_ efflux of bare soil (R_eb_, ppm min^−1^)^a^	2.945	7.824	19.934
Herbage contribution to CO_2_ efflux (R_ea_, ppm min^−1^)	0.256 (ns)	0.497	7.395
R-square (*p*)	0.198 (<0.0001)	0.604 (<0.0001)	0.596 (<0.0001)

The full model estimated CO_2_ efflux from bare soil and included terms for effect of air (or soil) temperature, the herbage contribution, and grazing intensity. Coefficients for simple quadratic and linear models of the air temperature effect on CO_2_ efflux are also presented.

a When replacing the term “air temperature” in the model.

b Entered into the model as grazing intensity: 0, 240 or 480 sheep ha^−1^ during grazing events for 0, 8 and 16 sheep ha^−1^ treatments, respectively.

c From Eq. 2 and assuming CO_2_ density of 1.902 kg m^−3^ at 280°K and 100 kPa, 1 ppm min^−1^ = 32.5 mg m^−2^ h^−1^ CO_2_ flux at 5°C and 67 kPa pressure typical of the Qinghai-Tibetan plateau. R_ea_ and R_eb_ denote above and below ground respiration, respectively.

A statistically significant positive correlation between grazing intensity and soil respiration was detected in summer (Table 2), but not winter or spring. Inclusion of a model term for hour of the day (results not presented) indicated a diurnal variation of intercept consistent with higher CO_2_ concentration at chamber closure during the night than during the day, but otherwise did not substantively change the results presented in Table 2.

**Figure 4:**
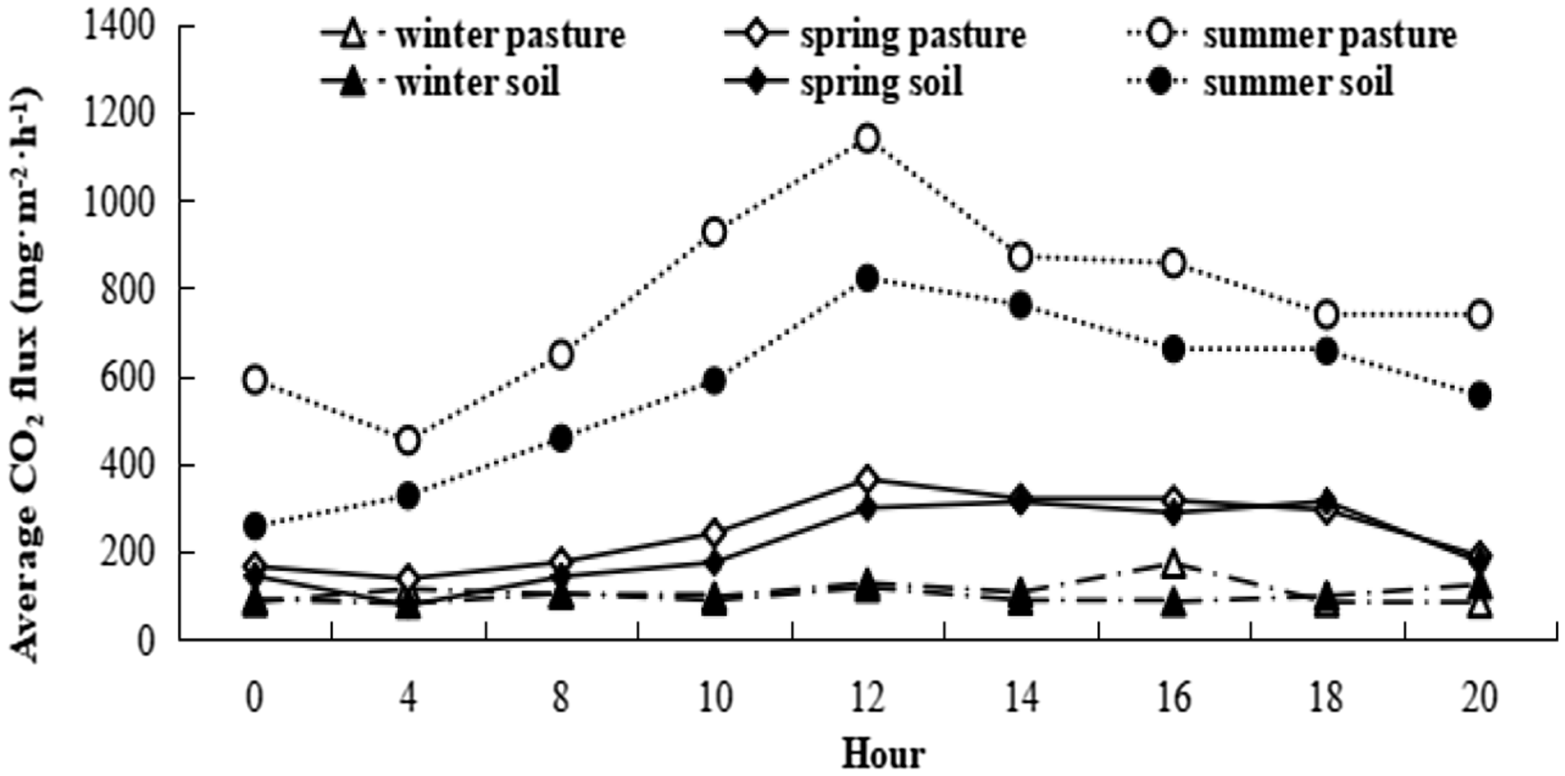
Seasonal variation in diurnal CO_2_ efflux for bare soil and intact vegetation.

Using coefficients in Table 2, soil respiration rates at this site without grazing and with air temperature at 5°C are estimated to be 223 and 307 mg CO_2_ m^−2^ h^−1^ in spring and summer, respectively, and 96 mg CO_2_ m^−2^ h^−1^ with soil and air temperatures at −5°C in winter. Herbage respiration increased CO_2_ emission (compared to bare soil) by 16 and 254 mg CO_2_ m^−2^ h^−1^ in spring and summer, respectively, but not unexpectedly was negligible in winter. The diurnal pattern of respiration for herbage and bare soil for the three measurement periods is shown in Figure 4. Notably, winter soil and herbage respiration fluxes were nonzero while spring fluxes more resembled those of winter than summer. The mean CO_2_ effluxes across the diurnal cycle measured for bare soil were 98, 214 and 566 mg CO_2_ m^−2^ h^−1^ in winter, spring and summer, respectively, while values for chambers with intact herbage were, respectively, 10, 31 and 209 mg CO_2_ m^−2^ h^−1^ greater than for bare soil. For comparison with other published studies, the seasonal values for R_e_ (i.e., R_ea_ + R_eb_) calculated from CO_2_ fluxes illustrated in Figure 4 were 3.9, 5.4, and 22.9 t CO_2_ ha^−1^ for winter, spring, and summer seasons, respectively, with the proportion of Re derived from R_eb_ being, respectively, 91%, 87%, and 73% in the same seasons. Herbage respiration was significantly increased on grazed plots, compared to ungrazed plots in summer only (Table 2).

### Herbage Mass at the Start and End of Grazing

**Figure 5:**
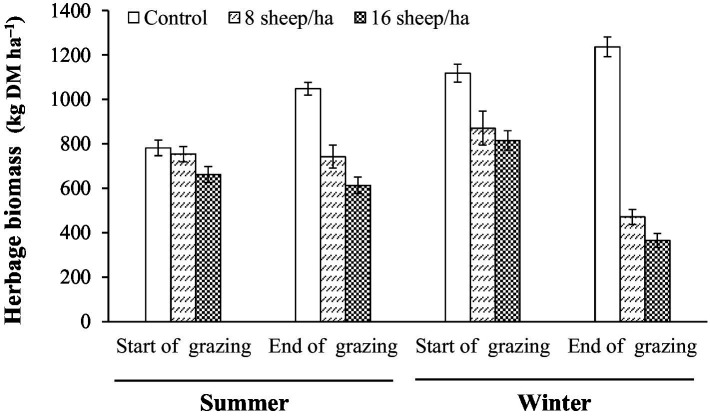
Herbage biomass at start and end of summer and winter grazing.

Herbage mass averaged approximately 750 kg DM ha^−1^ at the start of summer grazing and was approximately maintained under grazing but increased in the same period to a little over 1,000 kg DM ha^−1^ on Control plots. In winter-grazed plots herbage mass at start of grazing was similar to that in control plots at the end of summer grazing but was greatly reduced by grazing to approximately 400 kg DM ha^−1^ (Figure 5).

### Seasonal Data for Soil Microbial Biomass

Soil microbial biomass C values averaged approximately 2.5 g kg^−1^ dry soil with no observed response to grazing in the winter and summer measurements, but were higher and exhibited a grazing intensity response in spring (*p* = 0.015), ranging from 3.19 g kg^−1^ dry soil on control plots to 4.39 g kg^−1^ dry soil (SEM 0.39 g k^−1^ dry soil) in plots grazed at 16 sheep ha^−1^ (Table 3).

**Table 3:** Soil microbial biomass C and estimates for ecosystem carbon cycle components.

	Grazing intensity (sheep ha^−1^)^a^			
	0	8	16	SEM
*Soil microbial biomass C*				
Winter, November 2010 (g kg^−1^ dry soil)	2.60	2.19	2.95	0.40
Spring, April 2011 (g kg^−1^ dry soil)	3.19	4.08	4.39	0.39
Summer, August 2011 (g kg^−1^ dry soil)	2.80	2.52	2.67	0.31
*Winter grazing (1 October to 29 December 2010)*				
Est. herbage intake of sheep (t DM ha^−1^, 90 days)	−	639	1,281	123
Herbage mass change during grazing (kg DM ha^−1^)	122	−399	−450	124
Energy harvested (GJ ha^−1^ a^−1^)^b^	−	5.11	10.24	0.99
Metabolic grazing days (kg^0.75^ day ha^−1^ a^−1^)^c^	−	8,830	16,890	132
*Summer grazing (1 July to 30 September 2011)*				
Est. herbage intake of sheep (kg DM ha^−1^) (90 days)	−	380	730	68
Herbage mass change during grazing (kg DM ha^−1^)	266	−11	−108	61
Energy harvested (GJ ha^−1^)^b^	−	4.13	8.03	0.75
Metabolic grazing days (kg^0.75^ day ha^−1^ a^−1^)^c^	−	6,690	13,150	48
*Annual CO_2_ fluxes*				
Estimated CO_2_ loss by animal respiration (t ha^−1^ a^−1^)^d^	−	0.49	0.97	0.070
Total respiration (soil + herbage) (t CO_2_ ha^−1^ a^−1^)	28.0	29.5	33.4	1.3
Soil respiration (t CO_2_ ha^−1^ a^−1^)^e^	24.4	21.9	23.7	1.0
Herbage respiration (t CO_2_ ha^−1^ a^−1^)	3.6	7.6	9.7	1.6
Animal-induced summer respiration increase (t CO_2_ ha^−1^ a^−1^)^f^	−	0.075	0.149	0.024
Tentative estimate of herbage GPP (t CO_2_ ha^−1^ a^−1^)^g^	25.3	−	−	−

a Eight sheep on 1.0 or 0.5 ha, respectively, for 90 days with separate summer and winter plots.

b Calculations as described by Chen et al. (2010).

c Theoretically provides common units for feed demand of differing animal species to be summed.

d Assuming herbage 48% C and 55% of C ingested by animals respired (Parsons et al., 2013).

e Soil temperature response coefficients in this Table were used to adjust values downwards to correct for soil warming on bare soil with herbage removed in spring and summer and radiative cooling in winter.

f Calculated from co-efficient 0.0078 in Table 2.

g Calculated provisionally by combining data from two studies as described in “Scaling Up to Ecosystem Level and Impact of Grazing” section.

### Scaling Up to Ecosystem Level and Impact of Grazing

Calculated herbage removal by grazing animals was greater than the combined total of decline in herbage mass during grazing and herbage accumulation on ungrazed plots for both stocking rates in both summer and winter, indicating either reduced senescence of herbage on grazed plots or a possible stimulatory effect of grazing on herbage accumulation. The estimated energy yield of these grazing systems was a modest 5–10 GJ ha^−1^ a^−1^. Further investigation of the data used for the regression analysis in Table 2 showed that the increased CO_2_ efflux on grazed plots in summer was largely caused by grazing related increase in herbage respiration, not soil respiration (Table 3). From these data, the annualized estimate for soil + herbage respiration rate was 28.0 t CO_2_ ha^−1^ a^−1^, and the increase in respiration associated sheep grazing estimated by the model, while statistically significant, was a modest 0.075 and 0.149 t CO_2_ ha^−1^ for the summer period for the stocking rates of 8 and 16 sheep ha^−1^, respectively (Tables 2 and 3). Combining NEE data of Figure 5 in Kato et al. (2004b) for similar vegetation 60 km distant with our own R_ea_ and R_eb_ estimates we arrived at a tentative estimate of herbage GPP of 25.3 t CO_2_ ha^−1^ a^−1^ (Table 3).

## Discussion

### Seasonal Characteristics of Respiration Fluxes in Tibetan Plateau Alpine Meadow Pastures

Our study collected respiration data for intact vegetation and bare soil in ungrazed and grazed plots across three seasons in alpine meadows on the Qinghai-Tibetan Plateau in order to quantify seasonal and grazing-induced variation in soil and herbage respiration.

For winter, our salient finding was that CO_2_ efflux was not zero as might be intuitively supposed from the low winter temperatures, but was consistently positive, and amounted to 3.7 t CO_2_ ha^−1^ a^−1^ for the winter period. The fact that our regression model found no detectable herbage contribution to CO_2_ efflux in winter (Table 2) implies that plant leaf tissues were physiologically inactive, that photosynthesis in winter would also have been negligible, and root systems likely also dormant. This is also supported by Kato et al. (2004a) where it is shown that the temperature sensitivity (Q_10_) of CO_2_ efflux of a *Kobresia* meadow on the Qinghai-Tibetan Plateau showed a marked transition around −1.0°C. This suggests that winter R_eb_ activity may be coming mainly from microbial decomposition of soil C, rather than from plant root respiration. Subsequent to our study, others (e.g., Song et al., 2020) have confirmed the positive R_e_ values of alpine meadow pasture in winter and that grasslands on the Qinghai-Tibetan Plateau are CO_2_ sinks in the growing season and CO_2_ sources in winter.

Our data from spring are notable for the lag between the rise in R_ea_ and rise in R_eb_ as temperatures rose in spring. For example, mean soil temperatures over the winter, spring and summer measurement periods for which diurnal temperature curves are shown in Figure 2 were, respectively, −0.8, 6.4, and 16.5°C while model estimates (Table 2) of winter, spring and summer R_eb_ were, respectively, 2.8, 5.4, and 5.8 ppm CO_2_ min^−1^ (i.e., spring values were close to summer values). By contrast winter, spring and summer estimates of R_ea_ were, respectively, 0, 0.49, and 7.8 ppm CO_2_ min^−1^ (i.e., spring values were close to winter values). This implies that not even the whole growing season is available for carbon sequestration through leaf litter and root deposition to replace soil C respired during the comparatively long winter, and raises an interest in extending the current measurements to explore C balance factors in future research.

A relevant earlier study with which to compare our data is that of Kato et al. (2004b). These authors used the EC technique to determine NEE for an alpine meadow with botanical composition similar to that at our study site and in the same region. Two of four species listed as dominant by Kato et al. (2004b) (*Kobresia humilis* and *Kobresia tibetica*) were also present at our site and a third *Kobresia* species (*Kobresia graminifolia*) was one of the dominant species at our site (Sun et al., 2018). Kato et al. (2004b) note that the alpine *Kobresia* meadow ecosystem is one of the most widely distributed vegetation types on the vast Qinghai-Tibetan Plateau and occurs from 3,200 to 5,200 m altitude. They suggested that carbon budgets depend more on vegetation type than local geological conditions, so that their results may be taken as indicative for large areas of the Plateau. Their reporting focuses on diurnal fluxes and provides little clarity as to components of NEE identified in Eq. 1 above. They concluded from their EC NEE data that during the growing season from late May to the end of September there was a “small” net CO_2_ sequestration which we determined by extracting data from their Figure 5 to be 5.1 t CO_2_ ha^−1^.

It is interesting to note that the calculated winter R_eb_ from our study of 3.9 t CO_2_ ha^−1^ (apparently from oxidation of soil C as there was no herbage activity at this time), was only marginally smaller than the growing season sequestration of 5.1 t ha^−1^ recorded by Kato et al. (2004b) and that in late May those authors recorded very low daytime CO_2_ uptake, suggesting a herbage dormancy like that which we observed in our spring sampling in early May remained a feature of the ecosystem behavior at that point in the growing season. Further, neither our study nor that of Kato et al. (2004b) provide data for the months of April and October at the shoulders of the growing season, where based on present data there may also be a negative ecosystem carbon balance.

Another of our research objectives was elucidation of how R_ea_ and R_eb_ respond to imposition of sheep grazing. The only significant grazing effect on R_e_ detected by the regression analysis presented in Table 2 occurred in summer and further analysis (Table 3) showed that this effect primarily involved grazing stimulation of herbage respiration (R_ea_), with little change in R_eb_, and a tendency for grazing to decrease, rather than increase R_eb_. This finding was corroborated by comparatively small grazing-intensity-related changes in soil microbial biomass. Studies in North American rangeland (Thomas, 2012), Inner Mongolian steppe grassland (Tang et al., 2015), and a previous study in the Tibetan Plateau (Unteregelsbacher et al., 2011) have all reported reduced soil CO_2_ efflux under grazing, compared to ungrazed pastures, in common with the trend in our own data (Table 3).

A range of factors are likely involved in the apparent stimulation in herbage production under grazing and the associated summer increase in R_ea_. Firstly, the concentration of nutrients in dung and urine patches by grazing animals would have been expected to decrease soil respiration and increase herbage production (Wu et al., 2009; Kaštovská et al., 2010). Secondly, increased grazing intensity alters the temperature dependence of soil CO_2_ efflux, increasing soil CO_2_ efflux at a given temperature, and also increases soil temperature (Unteregelsbacher et al., 2011). Soil respiration response to increasing temperature should be enhanced at elevated temperatures (Yuste et al., 2009). The temperature sensitivity of organic matter decomposition decreases with increasing temperature, as indicated by the *Q*_10_ decreasing with temperature to be about 3.2 at 12°C and 1.4 at 24°C (Zhang et al., 2015), the logarithmic soil temperature-CO_2_ efflux relationship of Unteregelsbacher et al. (2011), and the significant quadratic relationship in the present study (Table 2; Figure 3C) also demonstrated this point. Thirdly, Fterich et al. (2012) demonstrated that microbial biomass of soil is maximal at low-to-intermediate levels of grazing influence and that the phenotypic evenness of the microbial community declines as the intensity of grazing increases, and soil microbial communities of heavily grazed sites are dominated by bacterial-based channels of decomposition. Lastly, a recent study of differentially expressed genes in *Stipa grandis* in steppe grassland in Inner Mongolia has shown that increased grazing pressure promotes expression of the Calvin-Benson cycle (Dang et al., 2021). Others have also noted that herbivory has a generally positive feedback effect, and can promote plant regrowth as well as energy and nutrient flows in grazed landscapes (Zhang Y. J. et al. 2014). All such effects on soil microbial dynamics feedback to soil respiration indirectly (Guo et al., 2012; Li et al., 2016).

### Implications for C-Balance and Sustainable Farming of Tibetan Alpine Meadow Pastures

A question of high topical interest with respect to R_ea_ and R_eb_ data like those from the present study, is whether or not there is evidence that increased grazing intensity of alpine meadows on the Qinghai-Tibetan plateau coupled with climate warming may be tipping these farming systems from C-sink to C-source status. In general herbivores consume plant tissue that would otherwise be cycled to the soil and exhale much of that directly to the atmosphere as CO_2_ from respiration. In addition, excreta returns are concentrated in small patches rather than distributed evenly across the grazed area, implying higher losses than when leaves senesce and die *in situ* (Parsons et al., 2013). Therefore, grazed plots might be expected to show a decline in soil C over time through lack of replenishment, compared to ungrazed plots. As noted above, Schlesinger and Andrews (2000) broadly predicted such effects, and Yuan and Hou (2015) documented evidence grazing-related soil C decline over time in deer pastures in the Qilian mountains. Alpine meadow soils of the Tibetan plateau typically contain about 7% C (Pei et al., 2008) with this high carbon content extending to 30–40 cm in depth. These soils are reported by Rui et al. (2011) to cover an area of about 110 million ha. Hence, there is potential for any change in their response to have a global impact on greenhouse gas accounting. A point of great interest at the present time is whether or not these soils remain a CO_2_ sink as they have been historically, or have become a net source.

Ultimately, such questions are likely to be answered by studies that incorporate both EC measurements of NEE, and direct measurements of one or more NEE components such as respiration or photosynthesis. Such studies are currently rare or non-existent because of logistical challenges. Our very tentative attempt to make such a calculation by combining our R_e_ data with NEE data extracted from Figure 5 of Kato et al. (2004b) and the estimate for GPP of 25.3 t CO2 ha^−1^ a^−1^ would indicate these ecosystems to now be CO_2_ sources, not CO_2_ sinks, but this estimate requires confirmation using data collected at the same site. Meanwhile, some comments are relevant on other approaches to determining C-balance of these ecosystems and issues that might arise based on findings of this study.

An approach, used by Wang et al. (2019) among others, is to estimate R_e_ from published relationships with soil temperature. These authors also did not conduct measurements in winter. However, it is clear from discussion in “Seasonal Characteristics of Respiration Fluxes in Tibetan Plateau Alpine Meadow Pastures” section above and from data of Figure 5 of Kato et al. (2004b) that in Tibetan Plateau *Kobresia* meadows there is a burst of herbage metabolic activity within a comparatively narrow time window during the months of April to October within the growing season so that activity predictions based on temperature, while they might capture soil microbial dynamics, would not capture associated herbage dynamics well. Hence, more data to define R_ea_ and R_eb_ and differentiate their heterotrophic and autotrophic components on the shoulders of the growing season in the months of May and October, in particular, are required for a complete understanding.

Another line of investigation for characterizing ecosystem source sink status is inventory of change in soil C content across time. Using this approach, Yang et al. (2010) concluded that soils on the Tibetan plateau have been carbon neutral for the last 20 years. Such results can be quite influential in causing redirection of research resources to other areas deemed a higher priority. However, this methodology would only detect accumulated change over time and would not be expected to detect an emerging trend in its early stages. Yang et al. (2010) indicated 127 ± 9 t ha^−1^ soil organic C to 100 cm, while the CO_2_ efflux indicated in Table 3, building linearly from zero over the 5 years prior to our study, would reduce soil C by about 7 t ha^−1^, or less than the measurement error for soil C in the study of Yang et al. (2010). Hence, their findings do not necessarily disprove the above conclusions that there is a concerning level of winter R_eb_ in these meadows, that may signal a shift toward a C-balance tipping point and will certainly be exacerbated by warming and any reduction in soil moisture associated with climate change.

There is also a precedent from a recent study of alpine soils in Europe, Rogiers et al. (2008) observed net annual CO_2_ efflux from alpine soils in Switzerland, and attributed the CO_2_ release to ongoing soil organic matter oxidation following drainage 40 years earlier. The Qinghai-Tibetan Plateau appears to be experiencing a similar soil-drying trend associated with the temperature increase indicated in Figure 1, despite there being no evidence of change in precipitation. For example, the total lake area of the Yamzhog Yumcoin southern Tibet decreased by about 67 km^2^ during 1980–1990 (Ye et al., 2008) and these authors concluded this was most likely a result of change in balance between precipitation and evaporation in the valley resulting from climate change. Study to determine if trends toward reduced soil moisture content might also be a factor determining soil-atmosphere CO_2_ fluxes on the Qinghai-Tibetan Plateau might also be rewarding.

### Livestock Carrying Capacity of the Environment

The above discussion leads us to conceptualize the Qinghai Tibetan Plateau farming systems as characterized by soils with a large historic accumulation of soil C from slow decomposition of returned above- and below-ground plant material in the prevailing cold climate. To develop sustainable farming practices in this context it is highly relevant to ask how much of the net primary production (NPP) can be harvested, and how much should be allowed to return to the soil to replace C lost by winter respiration, here indicated to be largely microbial oxidation of soil C. The above-ground NPP of *Kobresia* alpine meadows was stated by Zhang Q. et al. (2014) in their Table 1 to be about 1.5 t DM ha^−1^ a^−1^ and the recommended stocking rate is 1–5 animals ha^−1^. However, their discussion provides no clarification about classes or weights of animals and quantities of herbage removed at a particular stocking rate. Hence, in order to develop meaningful discussion on sustainable farming practices for the region it will be necessary to develop systems for recording animal numbers that correlate with animal feed demand. We provide further comment in “Methodology Considerations” section below on this point.

Intuitively, a grazing system similar to our eight sheep ha^−1^ grazing treatment with herbage removed estimated at 376 kg DM ha^−1^ with little change in herbage mass (Table 3) should be sustainable in the long term but more targeted data collection on herbage accumulation and animal intake through the growing season is required to confirm this. Our higher grazing intensity of 16 sheep ha^−1^ for 3 months would appear to have increased above-ground NPP (730 kg DM ha^−1^ herbage consumed with only 108 kg DM ha^−1^ decline in biomass, Table 3), but this may well have been at a cost to below-ground deposition. A crude estimate of reduction in litter deposition through removal of leaves before their senescence in summer grazed plots is obtained by adding the herbage mass reduction on grazed plots during the grazing season to the estimate of herbage eaten and comparing with herbage mass increase on Control plots (Table 3). Values obtained in this way are 655 kg DM ha^−1^ for the low stocking rate plots and 1,104 kg DM ha^−1^ for the high stocking rate plots. Inspection of Figure 5 shows that at the start of winter grazing in 2011 the herbage had not fully recovered from the previous year’s grazing activity and that the herbage mass reduction was greater during winter grazing than summer grazing. This is to be expected since in winter animals were heavier and their intake would have been enhanced by cold conditions, while herbage accumulation during grazing would have been less. Based on our data, we tentatively suggest that from an ecological sustainability perspective animal intake in grazed systems should be not more than 500 kg DM ha^−1^ a^−1^.

### Methodology Considerations

It has been pointed out (Li and Sun, 2011) that in the chamber method of measuring soil and herbage respiration, the elevated temperatures on bare soil after clipping may result in overestimation of soil respiration and underestimation of herbage respiration. We do not consider this would have been a problem in the current experiment for two reasons: Firstly the analysis presented in Table 2 considers the temperature inside the chamber during each measurement run as one of the variables affecting CO_2_ efflux rate; secondly we checked the data for evidence of change in CO_2_ efflux rate between successive 10 min intervals of chamber closure and found that any such trends in the data were usually small and not statistically significant. In fact, in our data for winter this “bare-soil-heating” effect following clipping was reversed. Bare soil tended to show reducing, not rising soil respiration rates with time since chamber closure in winter, indicating that clipped soil loses heat faster than a vegetation-covered surface in winter. In addition, in our calculations reported in Table 3, the CO_2_ fluxes were adjusted to correct for elevated temperature on bare soil.

The units of sheep stocking rate used here and based on body weight (kg^0.75^. grazing days; Table 3) will be unfamiliar to many readers. These units were chosen because body maintenance energy of an animal is proportional to (body weight)^0.75^, and hence units of kg^0.75^ animal body weight are widely used in farm systems research to represent the energy needs of different animal species in a single common formula (Ramírez-Restrepo et al., 2010). If future studies are reported in these units, it should be possible to compare data for animals of differing weight, and animal densities from different experiments using sheep, goats, yak, deer and other herbivores, without further adjustment. However, in such comparisons, factors such as pregnancy or body weight change during the experiment would need to be accounted for in the calculations in order to accurately estimate energy requirements of animals and herbage consumed. In that respect GJ ha^−1^ y^−1^ energy harvested from a grazing system is probably the ultimate unit of commonality when comparing systems and performing calculations to determine sustainability guidelines for graziers, once sustainable limitations have been experimentally determined. By contrast with the 4–10 GJ ha^−1^ a^−1^ reported in Table 3, more intensive temperate pastoral systems may yield energy contributions of over 150 GJ ha^−1^ a^−1^ to animal production (Matthew et al., 2010).

The estimation of herbage intakes of animals by feed budgeting based on their energy requirements underpins farm consultancy and industry regulation in Australasia and the United Kingdom and elsewhere. This is generally believed to be more accurate than direct measurement techniques like sampling of herbage to determine herbage disappearance at grazing or use of alkane markers (Narvaez et al., 2012). In fact, direct measurement of herbage intake tends to be resource intensive and often such data have a coefficient of variation >10%. The main requirement for use of energy budgeting methodology to infer herbage consumption by animals is recording of animal body weights at intervals during the experiment. Because collection of such data is not resource-intensive, data inferences are available in this study that have not been available from previous studies.

## Conclusion

Key insights emerging from our study of diurnal and seasonal CO_2_ efflux in an alpine meadow pasture in Maqu county on the Qinghai-Tibetan Plateau were: (1) soil respiration over winter was unexpectedly large. In sub-zero mean air temperatures of winter R_e_ continued at approximately 110 mg CO_2_ m^−2^ h^−1^. The winter respiration appears to be from microbial sources and not from plant roots. From a carbon accounting perspective the winter respiration and any warming-related increase is important as it tips the ecosystem from CO_2_ sink status toward CO_2_ source status; (2) herbage metabolic activity, as indicated by estimates of R_ea_ from our data was low in spring, indicating that substantive soil C sequestration by vegetation can happen only in a comparatively narrow time window within the growing season from June to September. More data on ecosystem CO_2_ fluxes for the months of May and September at the shoulders of the growing season are needed to gain a clear picture of annual C cycles; (3) decisions on stocking rate in farming systems on the Qinghai-Tibetan plateau should consider the need for return of plant litter to replace soil C lost through heterotrophic respiration; and (4) much greater clarity on the current source-sink status of these grasslands could be obtained if EC determinations of NEE were coupled with either direct measurements of components of R_e_ or direct measurements of GPP and NPP but initial indications from the current data are that warming temperatures and recent animal number increases may be tipping these alpine meadows to carbon-source status over an annual cycle.

## Data Availability Statement

The raw data supporting the conclusions of this article will be made available by the authors, without undue reservation.

## Author Contributions

FH: conceptualization, resources, project administration, and funding acquisition. FH, CM, XH, and HY: methodology. XH: software. FH, CM, and HY: validation. CM and XH: formal analysis. HY, YS, YL, TZ, XG, and CY: investigation. HY: data curation, writing—original draft preparation, and visualization. FH and CM: writing—review and editing. FH and JL: supervision. All authors contributed to the article and approved the submitted version.

## Funding

This research was financially supported by the National Natural Science Foundation of China, grant/award number: 32161143028 and U21A20242; the Program of National Science and Technology Assistance, grant/award number: KY202002011; the Program for Innovative Research Team of Ministry of Education, grant/award number: IRT17R50; and “Lanzhou City’s Scientific Research Funding Subsidy to Lanzhou University.” National Key Research and Development Program of China (2021YFD1300504).

## Conflict of Interest

The authors declare that the research was conducted in the absence of any commercial or financial relationships that could be construed as a potential conflict of interest.

## Publisher’s Note

All claims expressed in this article are solely those of the authors and do not necessarily represent those of their affiliated organizations, or those of the publisher, the editors and the reviewers. Any product that may be evaluated in this article, or claim that may be made by its manufacturer, is not guaranteed or endorsed by the publisher.
