# Supplementary material for: Seasonal Variation in Soil and Herbage CO2 Efflux for a Sheep-Grazed Alpine Meadow on the North-East Qinghai-Tibetan Plateau and Estimated Net Annual CO2 Exchange
Source: Front Plant Sci. 2022 Jun 2;13:860739. doi: 10.3389/fpls.2022.860739 (PMC9201780; doi:10.3389/fpls.2022.860739)
Supplement: Supplementary file 1 [file Table_1.docx]

Supplementary information:

**Table S1.** Linear regression of diurnal temperature ranges during gas flux measurement.

|  |  | Linear regression | R^2^ |
| --- | --- | --- | --- |
| Winter | Air Temp | y = -13.0254-1.8494x+0.5806x^2^-0.0244x^3^ | R^2^ = 0.8115 |
|  | Soil Temp | y = -1.9877-0.4282x+0.0919x^2^-0.0035x^3^ | R^2^ = 0.4142 |
| Spring | Air Temp | y = -2.1519-0.8695x+0.3948x^2^-0.0172x^3^ | R^2^ = 0.8396 |
|  | Soil Temp | y = 2.1714-1.8049x+0.3404x^2^-0.0121x^3^ | R^2^ = 0.8319 |
| Summer | Air Temp | y = 12.2278-2.4517x+0.4303x^2^-0.0150x^3^ | R^2^ = 0.7421 |
|  | Soil Temp | y = 2.1714-1.8049x+0.6353x^2^-0.0251x^3^ | R^2^ = 0.8106 |
